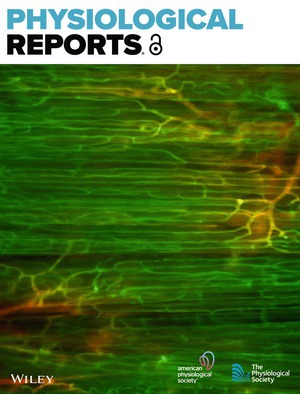# Cover Image

**DOI:** 10.14814/phy2.71046

**Published:** 2026-08-07

**Authors:** Mackenzie E. Charter, Coral L. Murrant

## Abstract

The cover image is based on the article *Capillary endothelial cell heterogeneity in skeletal muscle of female and male mice* by Coral Murrant et al., https://doi.org/10.14814/phy2.71025